# New Electroactive Polymers with Electronically Isolated 4,7-Diarylfluorene Chromophores as Positive Charge Transporting Layer Materials for OLEDs

**DOI:** 10.3390/molecules26071936

**Published:** 2021-03-30

**Authors:** Gintare Krucaite, Daiva Tavgeniene, Dovydas Blazevicius, Baohua Zhang, Aivars Vembris, Saulius Grigalevicius

**Affiliations:** 1 Department of Polymer Chemistry and Technology, Kaunas University of Technology, Radvilenu Plentas 19, LT50254 Kaunas, Lithuania; gintare.krucaite@ktu.lt (G.K.); daiva.tavgeniene@ktu.lt (D.T.); dovydas.blazevicius@ktu.lt (D.B.); 2 Center for Advanced Analytical Science, c/o School of Chemistry and Chemical Engineering, Guangzhou University, Guangzhou 510006, China; ccbhzhang@gzhu.edu.cn; 3 Laboratory of Organic Materials, Institute of Solid State Physics, University of Latvia, Kengaraga 8, LV-1063 Riga, Latvia; aivars.vembris@cfi.lu.lv

**Keywords:** polyether, fluorene, hole transporting material, ionization potential, organic light-emitting diode

## Abstract

A group of polyethers containing electroactive pendent 4,7-diarylfluorene chromophores have been prepared by the multi-step synthetic route. Full characterization of their structures has been presented. The polymeric materials represent derivatives of high thermal stability with initial thermal degradation temperatures in a range of 392–397 °C. Glass transition temperatures of the amorphous polymers range from 28 °C to 63 °C and depend on structures of the 4,7-diarylfluorene chromophores. Electron photoemission spectra of thin layers of the electroactive derivatives showed ionization potentials in the range of 5.8–6.0 eV. Hole injecting/transporting properties of the prepared polymeric materials were confirmed during formation of organic light-emitting diodes with tris(quinolin-8-olato)aluminium (Alq_3_) as a green emitter, which also serves as an electron transporting layer. The device using hole-transporting polymer with electronically isolated 2,7-di(4-biphenyl)fluorene chromophores demonstrated the best overall performance with low turn on voltage of 3 V, high current efficiency exceeding 1.7 cd/A, and with maximum brightness over 200 cd/m^2^. The organic light-emitting diode (OLED) characteristics were measured in non-optimized test devices. The efficiencies could be further improved by an optimization of device structure, formation conditions, and encapsulation of the devices.

## 1. Introduction

The advantages that organic light-emitting diode (OLED)-based technologies offer in terms of brightness, viewing angle, contrast ratio, production cost, opportunity for flexible displays, etc. are not rivaled by liquid crystal-based displays [1,2,3]. It is well established that multilayer devices comprising hole transport layer (HTL), electron transport layer, and emissive layer are necessary for efficient light emission [4,5,6].

One method that is very widely used to increase efficiencies of the organic light emitting devices is the incorporation of effective hole transporting layers in the structures of the OLEDs [1,7,8]. The charge transporting layers can be fabricated from low molecular weight compounds by vapor deposition or from polymeric materials by spin coating from prepared solutions. The solution-based route has the advantages, e.g., the production costs are lowered, large areas can be formed, and the molar mass of the materials is not limited [9,10].

The idea of attaching chromophoric fragments as the isolated side chain of a non-conjugated polymer was found to be of great advantage for the transformation of crystalline electroactive compounds to amorphous materials [11]. Advantageous aspects of this method are the availability of a great variety of functionalized amorphous polymers suitable for grafting reactions and high loading capacity without segregation of chromophores. Electro-optical properties could be also tuned by modification of molecular structure and by copolymerization [12].

Solution processed derivatives containing substituted carbazole fragments are among the most studied materials for organic electronics due to their good chemical and environmental stability as well as high positive charge mobility in their layers. In addition, derivatives containing electronically isolated carbazole chromophores have high triplet energies and are widely used as host materials for electro-phosphorescent devices (PhOLEDs) [13,14,15]. We have reported earlier that aryl substituted low-molar-mass carbazoles demonstrate suitable ionization potentials and positive charge transporting properties for application in OLED devices, however, the materials have evident tendency for crystallization [16]. Here, we report on polymeric hole transporting materials (HTM) containing diaryl-substituted fluorene fragments as electroactive chromophores. The materials are fully amorphous and were tested as hole transporting layers in OLEDs with Alq_3_ emitter demonstrating preparation of large area devices by spin-coating from solutions.

## 2. Results and Discussion

**Scheme 1:**
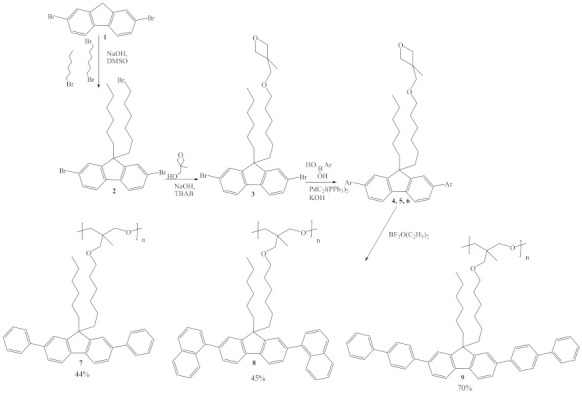
Ar = phenyl (monomer **4**), 1-naphtyl (monomer **5**), and 4-biphenyl (monomer **6**).

The synthesis of polymers **7**–**9** containing electroactive 2,7-diarylfluorenyl chromophores was carried out by a multi-step synthetic route as shown in Scheme 1. 2,7-Dibromo-9-(6-bromohexyl)-9-hexylfluorene (**2**) as a key material was synthesized from commercially available 2,7-dibromofluorene (**1**) by alkylation reaction with mixture of 1,6-dibromohexane and 1-bromohexane in DMSO. The derivative **2** was reacted under basic conditions with an excess of 3-methyl-3-oxetanemethanol to afford 2,7-dibromo-9-(6-[(3-methyloxetan-3-yl)methoxy]hexyl)-9-hexylfluorene (**3**). The monomers **4**–**6** were obtained from the compound **3** by Suzuki reaction with boronic acids, i.e., phenylboronic acid, 1-naphtalene boronic acid, or 4-biphenylboronic acid. Polymers **7**–**9** were prepared by cationic polymerization of the corresponding monomers **4**–**6** in 1,2-dichloretane solutions using BF_3_·O(C_2_H_5_)_2_ as an initiator. Low-molecular-weight fractions of the products of polymerizations were removed by Soxhlet extraction of the raw polymers with methanol.

All the newly synthesized derivatives were identified by IR and ^1^H NMR spectroscopy as well as by elemental analysis. Low-molar-mass compounds were also confirmed by mass spectrometry. The data were found to be in good agreement with the proposed structures. The polymers **7**–**9** were soluble in common organic solvents at room temperature. Amorphous thin films of these materials could be prepared by using cheap spin coating technique.

Molecular weights and polydispersity indices (PDI) of the prepared were estimated by GPC. The number-average molecular weights (M_n_), weight-average molecular weights (M_w_), and PDI of these polymers are presented in the Table 1. It was observed that rather low-molecular-weight polymeric materials were obtained after the cationic polymerization. It could be mentioned that the values of M_n_ and M_w_ of the polymers depend on the electronic structure of chromophores attached to the polymerizable unite. The monomer **4** containing 4,7-diphenylfluorene chromophores yielded polymer **7** with the lowest molecular weight and small PDI. It could be considered that polymer **7** has much better solubility due to smaller phenyl fragments at fluorene core and bigger amount of higher molecular weight fractions were removed during the Soxhlet extraction. The values of molecular weights of polyethers **8** and **9** containing 4,7-dinaphthylfluorene and 4,7-di(4-biphenyl)fluorene fragments were considerably higher. The polymers also have broad molecular weight distribution and big PDI values. It could be considered that the polymers having higher PDI values should demonstrate better film forming properties for formation of homogenous amorphous films. It could be also mentioned that molecular weights of polymers could be controlled by using other amount of initiator, by using other solvent for polymerization as well as by using other solvents for Soxhlet extraction of row polymer.

The behavior under heating of the polymeric materials **7**–**9** was tested under a nitrogen atmosphere by DSC and TGA methods. The synthesized polymers have very high thermal stabilities.

**Figure 1:**
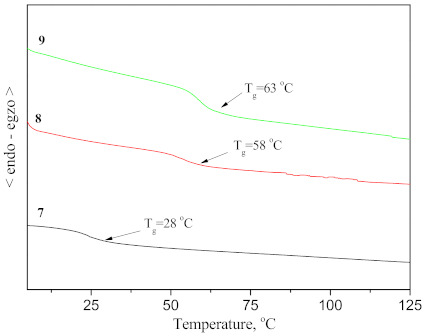
Differential scanning calorimetry (DSC) curves of polymers **7**–**9**. Heating rate was 10 °C/min.

DSC measurements have demonstrated that the synthesized polymers **7**–**9** are amorphous materials with clearly expressed glass-transition temperatures (T_g_). The curves of second heating of DSC measurements, which show the most exact T_g_ values of the polymers, are presented in Figure 1. It could be seen that the glass-transitions of the polymeric materials were observed at temperature of 28 °C for **7**, at 58 °C for **8**, and at 63 °C for **9**, and no peaks due to melting or crystallisation were obtained during all the measurements, i.e., the polymers are amorphous and suitable for preparation of thin homogeneous layers. It could be stated that T_g_ values of the materials depend on chemical compositions of chromophores of the polymers. The polymeric materials **8** and **9** with bulky biphenyl of naphtyl fragments have considerably higher T_g_ than that of polymer **7** having di-phenyl substituted fluorine core. The low T_g_ of the polymer 7 is a result of the aromatic chromophore of the material. Small mass and volume of the chromophore lead to low T_g_ value of the polymer.

**Figure 2:**
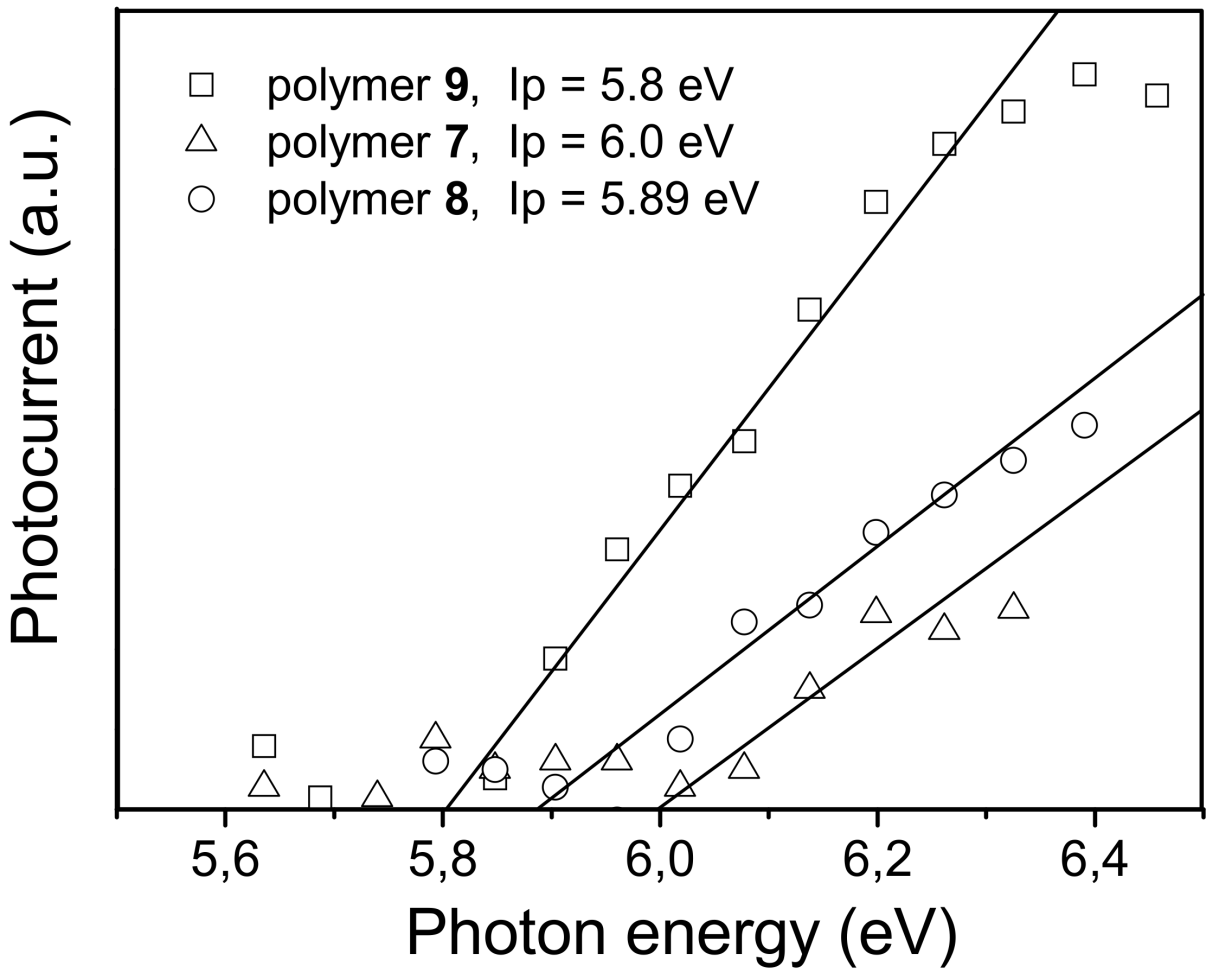
Electron photoemission spectra of thin layers prepared using the polymers **7**–**9**.

Thin amorphous layers of the polymers **7**–**9** were used for measurements of ionization potentials (I_p_) of these materials having various chromophores. The values of I_p_ were established from electron photoemission spectra of the thin films. The spectra and values of the I_p_ are shown in Figure 2. It could be observed that I_p_ of the materials range from 5.8 to 6.0 eV and depend on chemical compositions of the chromophores. The layer of polymer **8** having biphenyl-substituted fluorine fragments demonstrated the lowest I_p_ of 5.8 eV due to longer conjugated system in the aromatic fragments. I_p_ of the polymer **7** with biphenyl-substituted fluorine chromophores had the highest value, which reached 6.0 eV. The obtained characteristics show that the thin films of the prepared polymers should be suitable for hole injection and transport in OLED devices using emitters with high ionization potentials, i.e., deep highest occupied molecular orbital (HOMO) energy levels [17].

**Figure 3:**
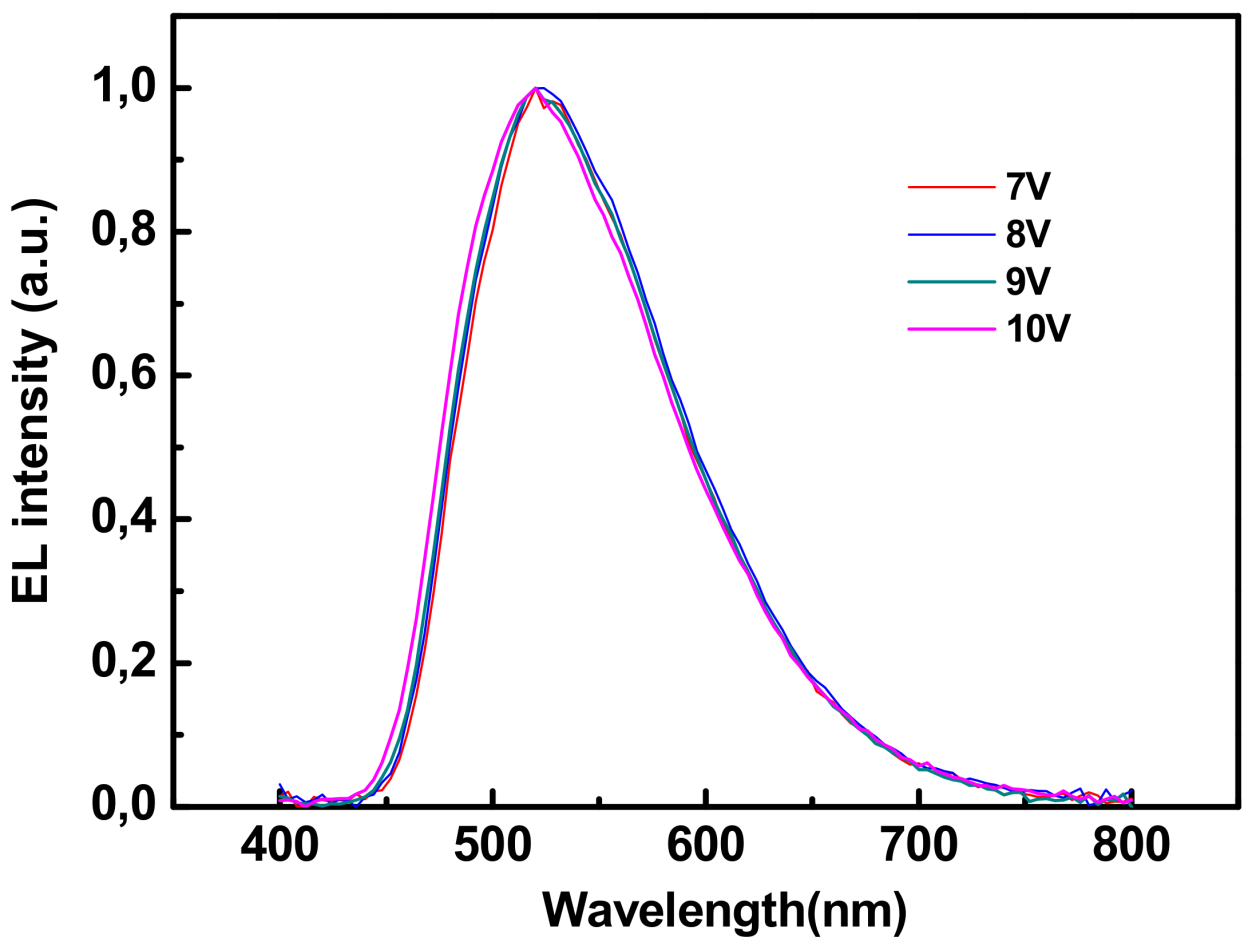
Electroluminescent spectra of the device indium tin oxide (ITO)/9/Alq_3_/LiF/Al at different voltages.

Positive charge transporting properties of layers of the prepared polymers **8**–**9** were tested in electroluminescent organic light-emitting diodes, which have been formed on glass substrates covered with ITO anode. It should be mentioned that polymer **7** could not compose homogenous thin layers suitable for the devices, probably due to its low glass transition temperature. Hole transporting layers of the polymeric materials **8** and **9** were prepared by spin-coating from their solution. Commercially available Alq_3_ was used as an emitter and also electron transporting layer in the OLEDs. Aluminium layer was used as a cathode in composition with a thin electron injection layer of LiF. When a positive voltage was applied for such OLEDs, a bright green electroluminescence originating from Alq_3_ emitting layer was obtained with an emission maximum at around 520 nm in all the devices. As an example, Figure 3 shows the electroluminescent spectra of OLED using polymer **9** as hole transporting layer (HTL) material at different voltages (7–10 V). This observation confirmed that the polymeric materials **8** and **9** only served as hole transporting layers without an exciplex formation at the interface with Alq_3_ emitter. The data also show that hole injection and mobility in the thin hole transporting films of the polymers was fully sufficient for an effective charge carrier recombination occurring within the Alq_3_ emitter layer.

**Figure 4:**
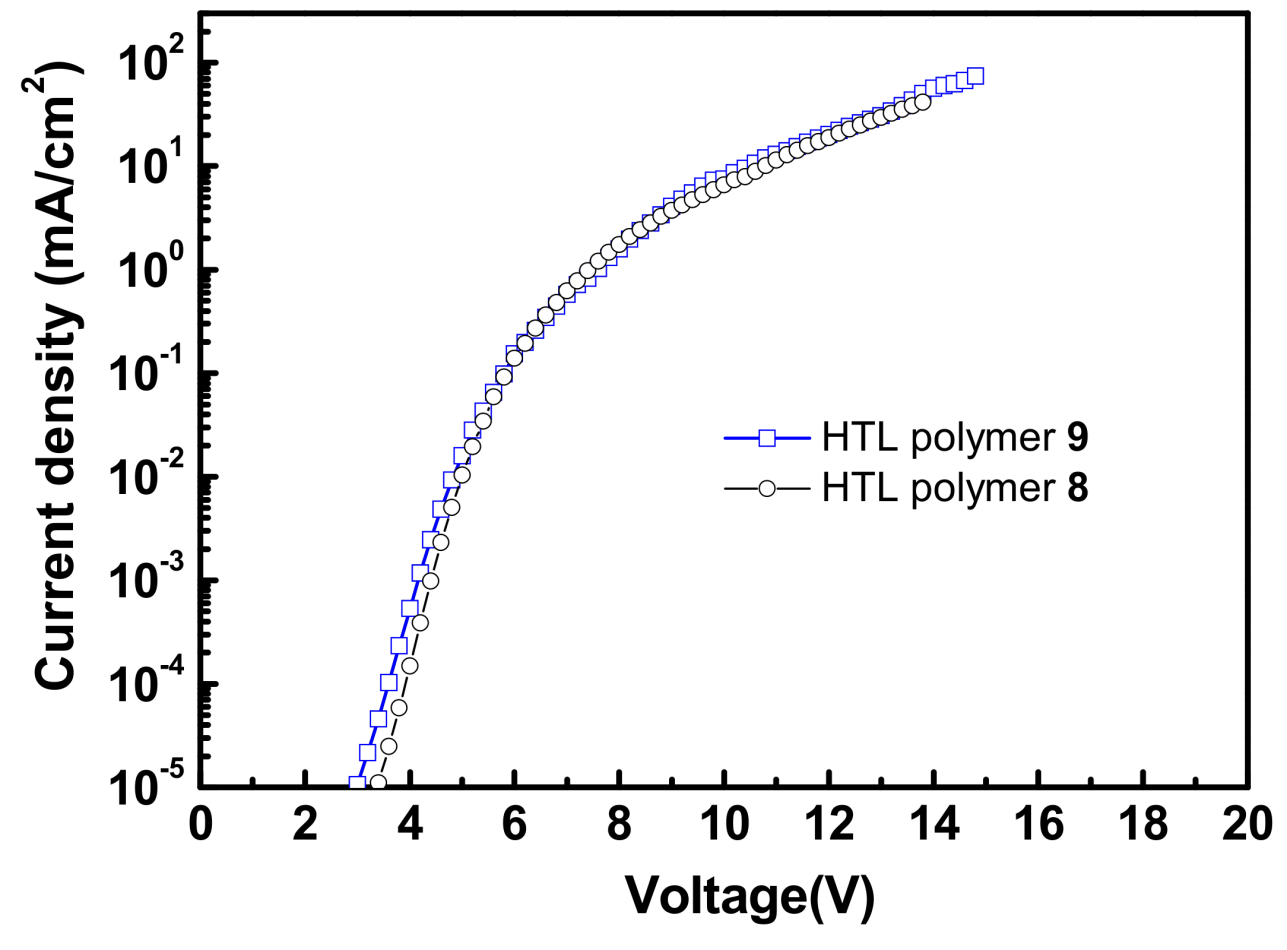

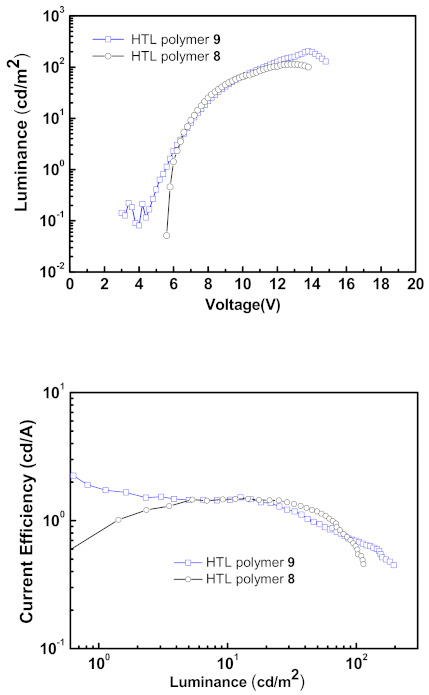
Organic light-emitting diode (OLED) characteristics of the devices with the configuration: ITO/**8** or **9**/Alq_3_/LiF/Al.

The current density–voltage (I–V) curves of the devices depicted in Figure 4 show a typical OLED’s behavior with rather low turn-on voltages of 3.0 V for diode using HTL of polymer **9** and of 5.6 V for device with HTL of **8**. The luminance–voltage and current efficiency–luminance characteristics for the OLEDs are also presented in the Figure 4. Both the devices could be characterized by maximum brightness of 113–204 cd/m^2^ at 12.5–13.5 V with current densities of 38–43 mA/cm^2^ and luminous efficiency of about 1.7 cd/A. The highest brightness exceeding 200 cd/m^2^ with efficiency of about 1.7 cd/A with was measured in the diode containing HTL of polymer **9** containing electro-active 2,7-di(4-biphenyl)fluorine chromophores. It was also observed from electron photoemission spectra that the materials were characterized by the lowest ionization potential, i.e., the best hole injecting properties. It should be pointed out that these OLED data were measured in non-optimized test devices. The efficiencies may be further improved by an optimization of device formation conditions, layer thicknesses as well as by encapsulation of the devices [18].

## 3. Experimental

### 3.1. Instrumentation

^1^H nuclear magnetic resonance (NMR) spectra were obtained using a Bruker Avance III (400 MHz) apparatus (Bruker, Berlin, Germany). The data are given as chemical shifts (δ) in ppm against trimethylsilane (in parenthesis: multiplicity, integration, coupling constant). Mass spectra were obtained on a Waters ZQ 2000 mass spectrometer (Agilent, Santa Clara, CA, USA). Infrared (IR) spectra are measured using a Vertex 70 Bruker spectrophotometer (Bruker, Berlin, Germany).

The molecular weights of the polymers were determined by a gel permeation chromatography (GPC) system Malvern/Viscotek (Malvern Panalytical, Westborough, MA, USA). Polystyrene standards were used for calibration of the columns and tetrahydrofuran (THF) was chosen as an eluent.

Differential scanning calorimetry (DSC) measurements were carried out using a Bruker Reflex II thermosystem (Bruker, Berlin, Germany). Thermogravimetric analysis (TGA) was performed on a TGAQ50 aparatus ((Bruker, Berlin, Germany)). The TGA and DSC curves were recorded in a nitrogen atmosphere at a heating rate of 10 °C/min.

The electron photoemission method for measurement of ionization potentials (I_p_) of the solid-state layers of the studied materials was exploited in air [19]. The spin-coated layers of the polymers onto commercial indium tin oxide (ITO)-coated glass substrates were utilized as the samples for the electron photoemission measurements. Thickness of the films for ionization potential measurements was 100 nm. The used experimental setup was the same as it was previously described [20].

The electroluminescent OLED devices were fabricated on glass substrates. The electro-active organic layers were sandwiched between a bottom indium tin oxide (ITO) anode and a top metal cathode. The ITO-coated substrates (Sigma-Aldrich) were carefully cleaned and treated with UV/ozone right before deposition of the organic layers. The substrates with a sheet resistance of 10 Ω/sq. and with ITO thickness of 150 nm were used. Hole transporting (HT) layers from the polymers were prepared by spin-coating from chloroform solutions (5 mg/mL). Thickness of the layers was controlled by changing speed (1100–1500 rpm for 60 s) of the substrate in order to obtain thin films of 40 nm. The thickness of the deposited polymer layer was measured by using a profilometer. Evaporation of the emitting/electron-transporting tris(quinolin-8-olato)aluminium (Alq_3_) layer (40 nm) and a LiF/Al electrode (1/150 nm) was done at the pressure of 1 × 10^−5^ torr in vacuum evaporation equipment. The final configuration of the devices was ITO/HT polymer/Alq_3_/LiF/Al. Size of the emitting areas was 0.10 cm^2^.

The luminance of the fabricated devices was measured using a Minolta CS-100 luminance-meter. A Keithley 2400 electrometer was used to measure the current-voltage characteristics of the devices. All the measurements were performed at ambient conditions in air.

### 3.2. Materials

2,7-Dibromofluorene (**1**), 1,6-dibromohexane, 1-bromohexane, tetra-n-butylammonium hydrogen sulfate (TBAHS),benzyltriethylammonium chloride, 3-methyl-3-oxetane methanol, tetrabuthylammonium bromide, 1-naphtalene boronic acid, phenylboronic acid, 4-biphenylboronic acid, bis(triphenylphosphine)palladium (II) dichloride (Pd(PPh_3_)_2_Cl_2_) boron trifluoride diethyl etherate (BF_3_·O(C_2_H_5_)_2_), and potassium hydroxide were purchased from Sigma Aldrich (München, Germany) and used as received.

2,7-Dibromo-9-(6-bromohexyl)-9-hexylfluorene (**2**) was obtained by a standard alkylation reaction of 2,7-dibromofluorene (**1**) using 1,6-dibromohexane and 1-bromohexane as the alkylation agents and KOH as the base [21,22]. A solution of 5 g (15.4 mmol) 2,7-dibromofluorene, 9.5 g (38.9 mmol) of 1,6-dibromohexane, and 6.35 g (38.4 mmol) of 1-bromohexane in tetrahydrofuran (100 mL) was stirred at 60 °C. Potassium hydroxide (2.6 g, 46.6 mmol), potassium carbonate (2 g, 15.2 mmol), and TBAHS (0.4 g, 1.1 mmol) were added to the solution. The reaction mixture was refluxed for 24 h. After TLC control, the reaction mixture was filtered. The crude product was purified by silica gel column chromatography using the mixture of ethyl acetate and hexane (vol. ratio 1:100) as an eluent. Yield: 3.4 g (39%) of viscous material. MS (APCI^+^, 20 V): 569 ([M + H], 100%). ^1^H NMR (400 MHz, CDCl_3_, δ, ppm): 7.52 (d, 2H, *J* = 1.4 Hz, Ar), 7.48–7.42 (m, 4H, Ar), 3.29 (tr, 2H, *J* = 11.9 Hz (CH_2_)_5_CH_2_Br), 1.97–1.83 (m, 4H, Al), 1.73–1.61 (m, 2H, Al), 1.23–1.00 (m, 10H, Al), 0.77 (tr, 3H, *J* = 12.6 Hz, Al), 0.63–0.53 (m, 4H, Al). Elemental analysis; found: C, 52.51; H, 5.49; molecular formula C_25_H_31_Br_3_ requires C, 52.57; H, 5.47; Br, 41.96%.

2,7-Dibromo-9-(6-[(3-methyloxetan-3-yl)methoxy]hexyl)-9-hexylfluorene (**3**) was obtained by alkylation reaction of 3-methyl-3-oxetane methanol with the 2,7-dibromo-9-(6-bromohexyl)-9-hexylfluorene (**2**) in the presence of NaOH base [21,22]. A solution of **2** (1 g, 1.79 mmol) and 3-methyl-3-oxetane methanol (0.28 g, 2.74 mmol) was stirred in 5 mL of toluene at 100 °C. There were 5 mL of sodium hydroxide solution (50%) in H_2_O and tetrabuthylammonium bromide (0.12 g, 0.04 mmol) was added to the solution. The reaction mixture was refluxed for 24 h. After TLC control, the reaction mixture was cooled. The product was extracted by using ethyl acetate and water. The combined organic extract was dried over anhydrous Na_2_SO_4_. The crude product was purified by silica gel column chromatography using the mixture of ethyl acetate and hexane (vol. ratio 1:15) as an eluent. Yield: 0.9 g (87%) of viscous material. MS (APCI^+^, 20 V): 591.14 ([M + H], 100%). ^1^H NMR (400 MHz, CDCl_3_, δ, ppm): 7.51 (d, 2H, *J* = 8.4 Hz, Ar), 7.46-7.43 (m, 4H, Ar), 4.46 (d, 2H, *J* = 5.6 Hz, CH_2_ of oxetanyl group), 4.32 (d, 2H, *J* = 5.6 Hz, CH_2_ of oxetanyl group), 3.40 (s, 2H, O-CH_2_-CH), 3.33 (tr, 2H, *J* = 7.0 Hz, CH_2_-O-CH_2_) 1.94–1.88 (m, 4H, Al), 1.42–1.36 (m, 2H, Al), 1.27(s, 3H, CH_3_), 1.15–1.00 (m, 10H, Al), 0.77 (tr, 3H, *J* = 7.0 Hz, Al CH_3_), 0.62–0.54 (m, 4H, Al). Elemental analysis; found: C, 60.78; H, 6.84; molecular formula C_30_H_40_Br_2_O_2_ requires C, 60.82; H, 6.81; Br, 26.97; O, 5.40%.

2,7-Diphenyl-9-(6-[(3-methyloxetan-3-yl)methoxy]hexyl)-9-hexylfluorene (**4**). There was 1 g (1.73 mmol) of 2,7-dibromo-9-(6-[(3-methyloxetan-3-yl)methoxy]hexyl)-9-hexylfluorene (**3**), 0.52 g (4.26 mmol) of phenylboronic acid, 0.05 g (0.071 mmol) of PdCl_2_(PPh_3_)_2_, and 0.5 g (8.91 mmol) of powdered potassium hydroxide were stirred in 15 mL of THF containing degassed water (1.5 mL) at 80 °C under nitrogen for 24 h. After TLC control the reaction mixture was cooled and quenched by the addition of ice water. The product was extracted by ethyl acetate. The combined extract was dried over anhydrous Na_2_SO_4_. The crude product was purified by silica gel column chromatography using the mixture of ethyl acetate and hexane (vol. ratio 1:5) as an eluent. Yield: 0.9 g (91%) of viscous material. MS (APCI^+^, 20 V): 587.38 ([M + H], 100%). ^1^H NMR (400 MHz, CDCl_3_, δ, ppm): 7.78 (d, 2H, *J* = 7.7 Hz, Ar), 7.69 (d, 4H, *J* = 8.4 Hz, Ar) 7.61–7.56 (m, 4H, Ar), 7.48 (tr, 4H, *J* = 7.7 Hz, Ar), 7.37 (tr, 2H, *J* = 7.7 Hz, Ar), 4.44 (d, 2H, *J* = 5.6 Hz, CH_2_ of oxetanyl group), 4.30 (d, 2H, *J* = 5.6 Hz, CH_2_ of oxetanyl group), 3.40 (s, 2H, O-CH_2_-CH), 3.31 (tr, 2H, *J* = 7.0 Hz, CH_2_-O-CH_2_), 2.09–2.03 (m, 4H, Al), 1.69–1.61 (m, 2H, Al), 1.24 (s, 3H, CH_3_), 1.15–1.03 (m, 10H, Al), 0.73–0.70 (m, 7H, Al ir Al CH_3_). Elemental analysis; found: C, 85.89; H, 8.61; molecular formula C_42_H_50_O_2_ requires C, 85.96; H, 8.59; O, 5.45%. FT-IR(KBr), cm^−1^: 3029, 2928, 2858, 1948, 1890, 1736, 1599, 1464, 1413, 1377, 1157, 1114, 978, 888, 826, 759, 696.

2,7-Di(1-naphtyl)-9-(6-[(3-methyloxetan-3-yl)methoxy]hexyl)-9-hexylfluorene (**5**). 1 g (1.73 mmol) of 2,7-dibromo-9-(6-[(3-methyloxetan-3-yl)methoxy]hexyl)-9-hexylfluorene (**3**), 0.75 g (4.36 mmol) of 1-naphtalene boronic acid, 0.05 g (0.071 mmol) of PdCl_2_(PPh_3_)_2_ and 0.5 g (8.91 mmol) of powdered potassium hydroxide were stirred in 15 mL of THF containing degassed water (1.5 mL) at 80 °C under nitrogen for 24 h. After TLC control the reaction mixture was cooled and quenched by the addition of ice water. The product was extracted by ethyl acetate. The combined extract was dried over anhydrous Na_2_SO_4_. The crude product was purified by silica gel column chromatography using the mixture of ethyl acetate and hexane (vol. ratio 1:10) as an eluent. Yield: 0.8 g (69%) of viscous material. MS (APCI^+^, 20 V): 687.41 ([M + H], 100%). ^1^H NMR (400 MHz, CDCl_3_, δ, ppm): 7.99 (d, 2H, *J* = 8.4 Hz, Ar), 7.95 (d, 4H, *J* = 8.4 Hz, Ar), 7.89 (tr, 4H, *J* = 7.7 Hz, Ar) 7.59–7.51 (m, 12H, Ar), 7.45 (tr, 2H, *J* = 8.4 Hz, Ar), 4.44 (d, 2H, *J* = 5.6 Hz, CH_2_ of oxetanyl group), 4.30 (d, 2H, *J* = 5.6 Hz, CH_2_ of oxetanyl group), 3.38 (s, 2H, O-CH_2_-CH), 3.34 (tr, 2H, *J* = 6.3 Hz, CH_2_-O-CH_2_) 2.07–2.00 (m, 4H, Al), 1.46–1.41 (m, 2H, Al), 1.24 (s, 3H, CH_3_), 1.21–1.09 (m, 10H, Al), 0.89–0.84 (m, 4H, Al), 0.80 (tr, 3H, *J* = 7.7 Hz, Al CH_3_). Elemental analysis; found: C, 87.38; H, 7.95; molecular formula C_50_H_54_O_2_ requires C, 87.42; H, 7.92; O, 4.66%. FT-IR(KBr), cm^−1^: 3057, 2927, 2857, 1928, 1738, 1508, 1457, 1392, 1267, 1159, 1113, 1006, 978, 828, 800, 777.

2,7-Di(4-biphenyl)-9-(6-[(3-methyloxetan-3-yl)methoxy]hexyl)-9-hexylfluorene (**6**). 1 g (1.73 mmol) of 2,7-dibromo-9-(6-[(3-methyloxetan-3-yl)methoxy]hexyl)-9-hexylfluorene (**3**), 0.86 g (4.34 mmol) of 4-biphenylboronic acid, 0.05 g (0.071 mmol) of PdCl_2_(PPh_3_)_2_, and 0.5 g (8.91 mmol) of powdered potassium hydroxide were stirred in 15 mL of THF containing degassed water (1.5 mL) at 80 °C under nitrogen for 24 h. After TLC control, the reaction mixture was cooled and quenched by the addition of ice water. The product was extracted by ethyl acetate. The combined extract was dried over anhydrous Na_2_SO_4_. The crude product was purified by silica gel column chromatography using the mixture of ethyl acetate and hexane (vol. ratio 1:7) as an eluent. Yield: 0.65 g (52%) of viscous material. MS (APCI^+^, 20 V): 739.44 ([M + H], 100%). ^1^H NMR (400 MHz, CDCl_3_, δ, ppm): 7.83–7.76 (m, 6H, Ar), 7.72 (d, 4H, *J* = 8.0 Hz, Ar), 7.70–7.63 (m, 8H, Ar), 7.49 (tr, 4H, *J* = 7.6 Hz, Ar), 7.38 (tr, 2H, *J* = 7.6 Hz, Ar), 4.44 (d, 2H, *J* = 5.6 Hz, CH_2_ of oxetanyl group), 4.29 (d, 2H, *J* = 5.6 Hz, CH_2_ of oxetanyl group), 3.37 (s, 2H, O-CH_2_-CH), 3.32 (tr, 2H, *J* = 6.8 Hz, CH_2_-O-CH_2_) 2.14–2.05 (m, 4H, Al), 1.89–1.82 (m, 2H, Al), 1.24 (s, 3H, CH_3_), 1.19–1.04 (m, 10H, Al), 0.81–0.68 (m, 7H, Al ir Al CH_3_). Elemental analysis; found: C, 87.71; H, 7.94; molecular formula C_54_H_58_O_2_ requires C, 87.76; H, 7.91; O, 4.33%. FT-IR(KBr), cm^−1^: 3059, 2925, 2858, 1926, 1741, 1508, 1455, 1393, 1266, 1157, 1115, 1011, 977, 826, 799, 775.

Poly{2,7-diphenyl-9-(6-[(3-methyloxetan-3-yl)methoxy]hexyl)-9-hexylfluorene} (**7**). Monomer **4** (0.9 g, 1.5 mmol) was polymerized in 3.1 mL of 1,2-dichlorethane by using 6.5 mg (5.65 µL, 0.046 mmol) of BF_3_·O(C_2_H_5_)_2_ as an initiator. Polymerization mixture was stirred at 60 °C under nitrogen for 24 h. After precipitation into methanol, the low molecular weight fractions of the polymer were removed by Soxhlet extraction of the raw material with methanol. Yield: 0.4 g (44%) of white amorphous powder. ^1^H NMR (400 MHz, CDCl_3_, δ, ppm): 8.01–7.70 (m, 6H, Ar), 7.56–7.30 (m, 10H, Ar), 3.21–2.92 (m, 6H, Al), 2.17 (s, 2H, OCH_2_CH), 2.05–1.83 (m, 4H, Al), 1.39–1.21 (m, 10H, Al), 1.19–0.94 (m, 8H, Al), 0.91–0.70 (m, 10H, Al). Elemental analysis; found: C, 85.87; H, 8.63; molecular formula (C_42_H_50_O_2_)_n_ requires C, 85.96; H, 8.59; O, 5.45%. FT-IR(KBr), cm^−1^: 3007, 2928, 2857, 2797, 1713, 1599, 1464, 1393, 1177, 1114, 981, 882, 825, 759, 696.

Poly{2,7-di(1-naphtyl)-9-(6-[(3-methyloxetan-3-yl)methoxy]hexyl)-9-hexylfluorene} (**8**). Monomer **5** (0.7 g 1.0 mmol) was polymerized in 2.1 mL of 1,2-dichlorethane by using 4.3 mg (3.74 µL, 0.036 mmol) of BF_3_·O(C_2_H_5_)_2_ as an initiator. Polymerization mixture was stirred at 60 °C under nitrogen for 24 h. After precipitation into methanol, the low molecular weight fractions of the polymer were removed by Soxhlet extraction of the raw material with methanol. Yield: 0.45 g (64%) of white amorphous powder. ^1^H NMR (400 MHz, CDCl_3_, δ, ppm): 7.80–7.21 (m, 20H, Ar), 3.28–2.92 (m, 6H, Al), 2.17 (s, 2H, OCH_2_CH), 2.08–1.92 (m, 4H, Al), 1.40–1.17 (m, 4H, Al), 1.13–0.95 (m, 8H, Al), 0.89–0.60 (m, 10H, Al). Elemental analysis; found: C, 87.35; H, 7.98; molecular formula (C_50_H_54_O_2_)_n_ requires C, 87.42; H, 7.92; O, 4.66%. FT-IR(KBr), cm^−1^: 3459, 3057, 2927, 2854, 1634, 1456, 1392, 1375, 1268, 1099, 1050, 1015, 800, 777.

Poli{2,7-di(4-biphenyl)-9-(6-[(3-methyloxetan-3-yl)methoxy]hexyl)-9-hexylfluorene} (**9**). Monomer **6** (0.5 g 0.68 mmol) was polymerized in 1.4 mL of 1,2-dichlorethane by using 2.8 mg (2.43 µL, 0.02 mmol) of BF_3_·O(C_2_H_5_)_2_ as an initiator. Polymerization mixture was stirred at 60 °C under nitrogen for 24 h. After precipitation into methanol, the low molecular weight fractions of the polymer were removed by Soxhlet extraction of the raw material with methanol. Yield: 0.35 g (70%) of white amorphous powder. ^1^H NMR (400 MHz, CDCl_3_, δ, ppm): 7.81–7.23 (m, 24H, Ar), 3.22–2.85 (m, 10H, Al), 2.17 (s, 2H, OCH_2_CH), 2.09–1.89 (m, 4H, Al), 1.15–0.91 (m, 10H, Al), 0.81–0.59 (m, 8H, Al). Elemental analysis; found: C, 87.69; H, 7.95; molecular formula (C_54_H_58_O_2_)_n_ requires C, 87.76; H, 7.91; O, 4.33%. FT-IR(KBr), cm^−1^: 3460, 3028, 2927, 2852, 1907, 1634, 1484, 1464, 1401, 1362, 1246, 1099, 1004, 888, 817, 763, 694.

## 4. Conclusions

In conclusion, new electro-active polymers having 4,7-diarylfluorene chromophores were prepared by cationic polymerization of the corresponding oxetane-based monomers. The amorphous materials demonstrated very high thermal stability (392–397 °C) and glass transition temperatures in a range of 28–63 °C. Electron photoemission spectra of thin films of the polymers confirmed that ionization potentials of the materials are depending on their chromophores and are in a range from 5.8 eV to 6.0 eV. The potentials could be suitable for hole injection and transport into emitting layers having high ionization potentials. The polymers have been tested as positive charges transporting films in bilayer organic light-emitting diodes with tris(quinolin-8-olato)aluminium (Alq_3_) as an emitter as well as electron transporting layer. An OLED device with polymer having electro-active 2,7-di(4-biphenyl)fluorine chromophores exhibited the best overall performance with low turn on voltage of 3 V, maximum brightness exceeding 200 cd/m^2^, and current efficiency of 1.7 cd/A. These OLED properties are rather promising among Alq_3_-based two-layer devices having polymeric hole transporting layers.

## Figures and Tables

**Table 1:** Molecular weights and polydispersity indices (PDI) of the oligomers **7**–**9**.

Polymer	M_n_	M_w_	PDI
**7**	2730	3690	1.35
**8**	3930	8210	2.09
**9**	6720	17,640	2.63

## Data Availability

The data presented in this study are available on request from the corresponding author.
